# Artificial intelligence in imaging diagnosis of liver tumors: current status and future prospects

**DOI:** 10.1007/s00261-025-05059-8

**Published:** 2025-06-19

**Authors:** Masatoshi Hori, Yuki Suzuki, Keitaro Sofue, Junya Sato, Daiki Nishigaki, Miyuki Tomiyama, Atsushi Nakamoto, Takamichi Murakami, Noriyuki Tomiyama

**Affiliations:** 1 Department of Artificial Intelligence in Diagnostic Radiology, The University of Osaka Graduate School of Medicine, 2-2, Yamadaoka, 565-0871 Suita, Japan; 2 Department of Radiology, Kobe University Graduate School of Medicine, 7-5-2 Kusunoki-cho, Chuo-ku, 650-0017 Kobe, Japan; 3 Department of Diagnostic and Interventional Radiology, The University of Osaka Graduate School of Medicine, 2-2, Yamadaoka, 565-0871 Suita, Japan

**Keywords:** Artificial intelligence, Liver tumors, Liver neoplasms, Medical imaging, Hepatocellular carcinoma, Radiomics

## Abstract

Liver cancer remains a significant global health concern, ranking as the sixth most common malignancy and the third leading cause of cancer-related deaths worldwide. Medical imaging plays a vital role in managing liver tumors, particularly hepatocellular carcinoma (HCC) and metastatic lesions. However, the large volume and complexity of imaging data can make accurate and efficient interpretation challenging. Artificial intelligence (AI) is recognized as a promising tool to address these challenges. Therefore, this review aims to explore the recent advances in AI applications in liver tumor imaging, focusing on key areas such as image reconstruction, image quality enhancement, lesion detection, tumor characterization, segmentation, and radiomics. Among these, AI-based image reconstruction has already been widely integrated into clinical workflows, helping to enhance image quality while reducing radiation exposure. While the adoption of AI-assisted diagnostic tools in liver imaging has lagged behind other fields, such as chest imaging, recent developments are driving their increasing integration into clinical practice. In the future, AI is expected to play a central role in various aspects of liver cancer care, including comprehensive image analysis, treatment planning, response evaluation, and prognosis prediction. This review offers a comprehensive overview of the status and prospects of AI applications in liver tumor imaging.

## Introduction

In 2020, liver cancer was the 6th most commonly diagnosed cancer worldwide and the 3rd leading cause of cancer-related deaths. Approximately 905,700 new cases and 830,200 deaths were reported globally. By 2040, these numbers are projected to increase by > 55%, reaching an estimated 1.4 million new cases and 1.3 million deaths annually [1]. Medical imaging techniques—such as ultrasound, computed tomography (CT), magnetic resonance imaging (MRI), and positron emission tomography (PET)—play crucial roles in diagnosing and managing liver tumors, including hepatocellular carcinoma (HCC) and metastatic liver cancer. These imaging techniques are used for surveillance, detection, diagnosis, staging, treatment planning, and post-treatment follow-up. Advances in imaging modalities and evaluation methods have greatly contributed to the management of patients with liver tumors. Since 2011, the Liver Imaging Reporting and Data System (LI-RADS) has evolved as a comprehensive system to standardize imaging practices in patients with liver cirrhosis or chronic hepatitis B [2, 3].

Despite significant advancements, the imaging diagnosis of liver tumors still presents numerous challenges. Accurate diagnosis using CT or MRI is still challenging for detecting small HCCs, because of its low sensitivity [4]. Moreover, liver imaging includes multi-phase, three-dimensional (3D) volumetric and functional imaging using liver-specific contrast agents in MRI. Therefore, the amount of information generated via these techniques tends to be substantial. This underscores the need for skilled abdominal radiologists who specialize in liver imaging. However, such specialists may not always be available at the clinical site, making it difficult to properly evaluate liver images. There are also challenges associated with interpretation using LI-RADS. The LI-RADS system presents notable challenges, including the substantial reporting burden imposed by its highly detailed templates, which require explicit documentation of imaging features, and the intrinsic subjectivity in interpreting radiologic findings, which contributes to interreader variability even in seemingly objective measures such as lesion size [3]. Many researchers expect that artificial intelligence (AI), which has made significant progress in recent years, will help address these challenges.

AI has been explored since the 1950s as an attempt to simulate human-like cognitive processes using machines. Since the advent of deep learning in 2012, AI has rapidly gained prominence across various fields, including medicine. Deep learning shows remarkable progress in image recognition and classification, and its application in radiology began early. Currently, AI is applied in areas such as lesion detection, tumor characterization, segmentation (of organ, anatomical region, or lesion boundaries), and image quality enhancement, which support radiation dose reduction or shorten imaging time. In addition, AI is increasingly used in radiomics, with ongoing research exploring its potential to predict treatment outcomes and support other clinical applications. Earlier studies have reported on the application of AI in detecting and characterizing liver tumors [5]. This review aims to examine the current status and prospects of AI in liver tumor imaging.

### Deep learning reconstruction

**Fig. 1:**
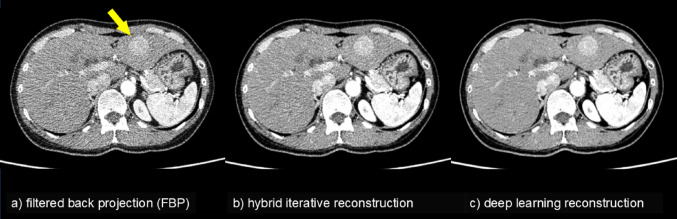
Contrast-enhanced CT images (late arterial phase, ultra-high-resolution CT with a 1024 matrix and 0.25 mm thickness, reconstructed from the same acquisition data using three different methods, including deep learning reconstruction). Traditionally, CT images use a 512 × 512 matrix size, but with ultra-high-resolution CT equipment (Aquilion Precision, Canon Medical Systems), it allows for images with a 1024 × 1024 matrix size. Higher resolution generally increases image noise. **a** Using the conventional FBP method, a hypervascular nodule of hepatocellular carcinoma is depicted in the lateral segment of the left liver lobe (arrow), but its visibility is poor owing to significant image noise. **b** With the more recent mainstream hybrid iterative reconstruction methods (AIDR 3D; Canon Medical Systems), the visibility of the lesion has improved. **c** Using deep learning reconstruction (AiCE; Canon Medical Systems), image noise is significantly reduced compared to that of **b**), and lesion visibility is further enhanced. Deep learning reconstruction is useful for improving image quality, enhancing lesion depiction, and reducing radiation exposure. *CT* Computed tomography, *FBP* Filtered back projection

Image reconstruction is the process of creating cross-sectional images from raw data obtained via signals, such as X-rays or electromagnetic waves. Application of AI to this process can enhance image quality in medical imaging techniques such as CT and MRI. Currently, major manufacturers offer diagnostic imaging scanners equipped with deep learning reconstruction (DLR) techniques. While the specifics of DLR differ by manufacturer, the general principle involves incorporating AI trained on high‒quality images to reconstruct superior-quality images from raw data. DLR typically produces images with reduced noise (Fig. 1). As discussed below, the advent of DLR has contributed to improved image quality and diagnostic accuracy, reduced radiation exposure in CT, and shortened scan times in MRI.

**Fig. 2:**
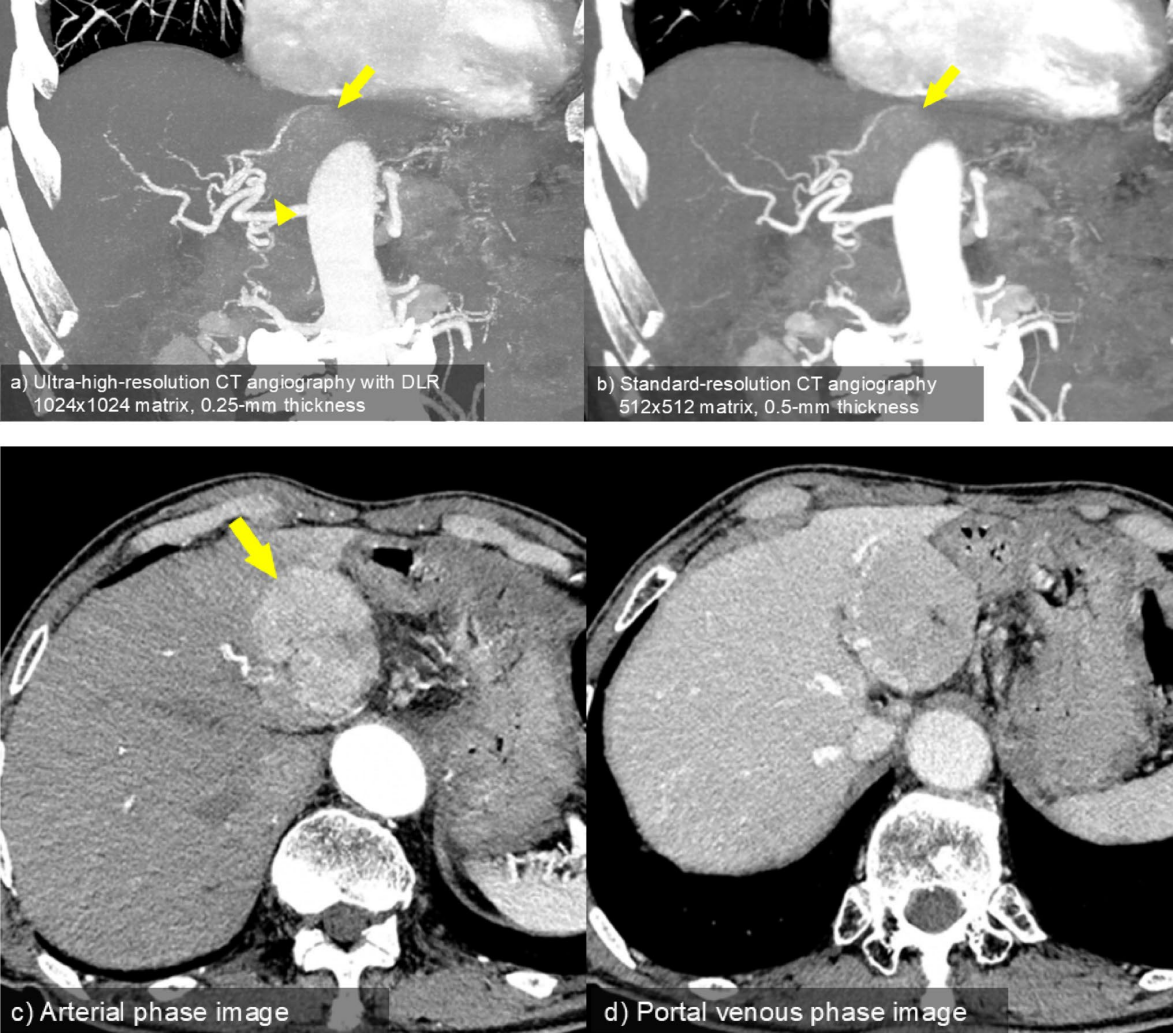
Utility of ultra-high-resolution CT angiography with DLR in treatment planning for HCC. **a** Ultra-high-resolution CT angiography (MIP image) with a 1024 × 1024 matrix, reconstructed using a DLR algorithm (AiCE; Canon Medical Systems). **b** Standard-resolution CT angiography (MIP image) with a 512 × 512 matrix, reconstructed from the same raw data. Transverse images during (**c**) arterial and (**d**) portal venous phases. Traditionally, CT images use a 512 × 512-matrix size, but with ultra-high-resolution CT equipment (Aquilion Precision, Canon Medical Systems) it enables images with a 1024 × 1024-matrix size. A hypervascular tumor is visible (arrows). Ultra-high-resolution CT angiography using DLR provides superior delineation of small hepatic artery branches (arrowhead) compared to that of standard resolution. The improved image clarity provided by DLR in ultra-high-resolution CT angiography aids in detailed assessment of vascular anatomy, essential for planning transarterial chemoembolization therapy in patients with HCC. *CT* Computed tomography, *DLR* Deep learning reconstruction, *HCC* Hepatocellular carcinoma, *MIP* Maximum Intensity Projection

Obtaining high spatial resolution images in both CT and MRI generally increases image noise. Efforts to reduce X-ray radiation exposure during CT and shortened MRI scan times often result in increased image noise. Conversely, suppressing noise with DLR can enhance spatial resolution (Fig. 2). For high-resolution abdominal CT, it has been reported that when images were reconstructed from the same raw data using DLR, hybrid iterative reconstruction (IR), and model-based IR, the image noise was significantly lower and the contrast-to-noise ratio was significantly higher with DLR compared to both hybrid IR and model-based IR [6].

In liver CT, where the contrast between tumors and surrounding organ parenchyma is lower than that of lung CT, minimizing image noise is crucial for tumor detection. AI-driven noise reduction can thus improve liver tumor detectability. A recent study demonstrated that, compared to conventional hybrid IR, DLR significantly improved the detection performance of HCC, interobserver agreement in LI-RADS categorization, and overall image quality in dynamic contrast-enhanced CT. The figure of merit for HCC detection was significantly higher with DLR than with hybrid IR, and DLR also yielded significantly better interobserver agreement [7]. An additional study similarly reported that DLR outperformed hybrid IR in LI-RADS categorization and reader confidence [8]. Evidence from another investigation indicated that DLR significantly reduced noise relative to IR, without affecting noise texture, which may aid in detecting hypervascular liver lesions on dual-energy CT [9]. In addition to its utility for HCC, DLR has also been shown to improve the detection of liver metastases, offering better lesion conspicuity and higher sensitivity compared to IR [10].

Studies have demonstrated that DLR reduces radiation exposure in CT while maintaining image quality. For instance, a recent multicenter study demonstrated that low-dose CT using only 33% of the standard radiation dose, when combined with DLR, achieved lower image noise and non-inferior diagnostic performance for detecting malignant liver tumors compared to standard-dose CT with model-based IR [11]. Another study also reported a 34% dose reduction with DLIR while maintaining detection performance for liver metastases [12].

DLR is also useful for reducing scan time in MRI. For example, a recent study demonstrated that deep learning–accelerated multi-b-value diffusion-weighted MRI reduced acquisition time by over 50%, improved image quality, and maintained predictive performance for microvascular invasion in HCC [13]. An abbreviated MRI protocol using DLR has been shown to enhance image quality and reduce scan time by 50%, while preserving diagnostic sensitivity for malignant liver lesions [14].

Additionally, AI-based super-resolution techniques, which generate high‒resolution images from lower‒resolution data, are expected to allow for more detailed evaluation of small structures [15].

### Detection of liver tumors using AI

Researchers have studied AI systems capable of detecting tumors from medical images [5]. High-performance AI tools for lesion detection in chest X-rays and CT scans have been developed and are becoming increasingly integrated into clinical practice. In liver imaging, progress remains slower compared to that of chest imaging. Several factors contribute to this challenge: (a) the contrast between lesions and surrounding tissues in liver CT is significantly lower than that in chest CT; (b) dynamic multiphasic imaging plays a critical role in liver CT, making the processing more complex; and (c) liver MRI requires the interpretation of several image types [16]. These are key challenges that must be addressed for AI-based liver tumor detection systems to become well-established in clinical practice.

However, recent years have seen promising advances. Advances in AI-based software for liver imaging have shown encouraging results. In one study using contrast-enhanced MRI data from 395 patients, AI demonstrated a detection sensitivity of 0.848 for lesions < 20 mm—outperforming radiologists—and achieved equivalent performance for larger lesions [17]. Combined interpretation further improved overall sensitivity to 0.883. Lesion size measurements by AI aligned well with pathology (*P* = 0.174), and the average segmentation Dice coefficient was 0.62, supporting the role of AI as a reliable adjunct in liver MRI assessment.

Recent research suggests that AI can help reduce the rate of missed liver tumors on diagnostic imaging. One study investigated the effectiveness of AI-powered software in detecting liver metastases that had been overlooked on contrast-enhanced CT [18]. Among 135 analyzable cases, the software demonstrated a per-lesion sensitivity of 70.8% for all metastases and 55.0% for lesions missed by radiologists. It identified metastases in 53.7% of overlooked cases, with an average of only 0.48 false positives per patient. These findings indicate that AI has the potential to meaningfully improve lesion detection when integrated into radiologic workflows. In another study, researchers assessed the feasibility and efficiency of using AI to identify missed incidental suspicious liver lesions on 2,573 CT pulmonary angiographic examinations [19]. AI algorithms flagged 136 potential cases, of which 13 were confirmed as true misses (0.5%) following radiologist review. The AI-assisted workflow achieved a 10:1 review-to-yield ratio, compared to a baseline estimate of 66:1 without AI. These results support the role of AI in reducing radiologist workload while maintaining diagnostic accuracy.

### Characterization of liver tumors using AI

Attempts to differentiate hepatic lesions on liver CT using deep learning began soon after the technology emerged [5]. In 2018, convolutional neural networks (CNN), a type of deep learning model, demonstrated effectiveness in differentiating liver tumors on dynamic CT scans [20]. Recently, AI systems that combine liver CT with clinical data—such as sex, age, total bilirubin levels, and tumor markers—have also been developed for tumor classification, using a combination of deep CNNs and gated recurrent neural networks (RNNs) [21].

**Fig. 3:**
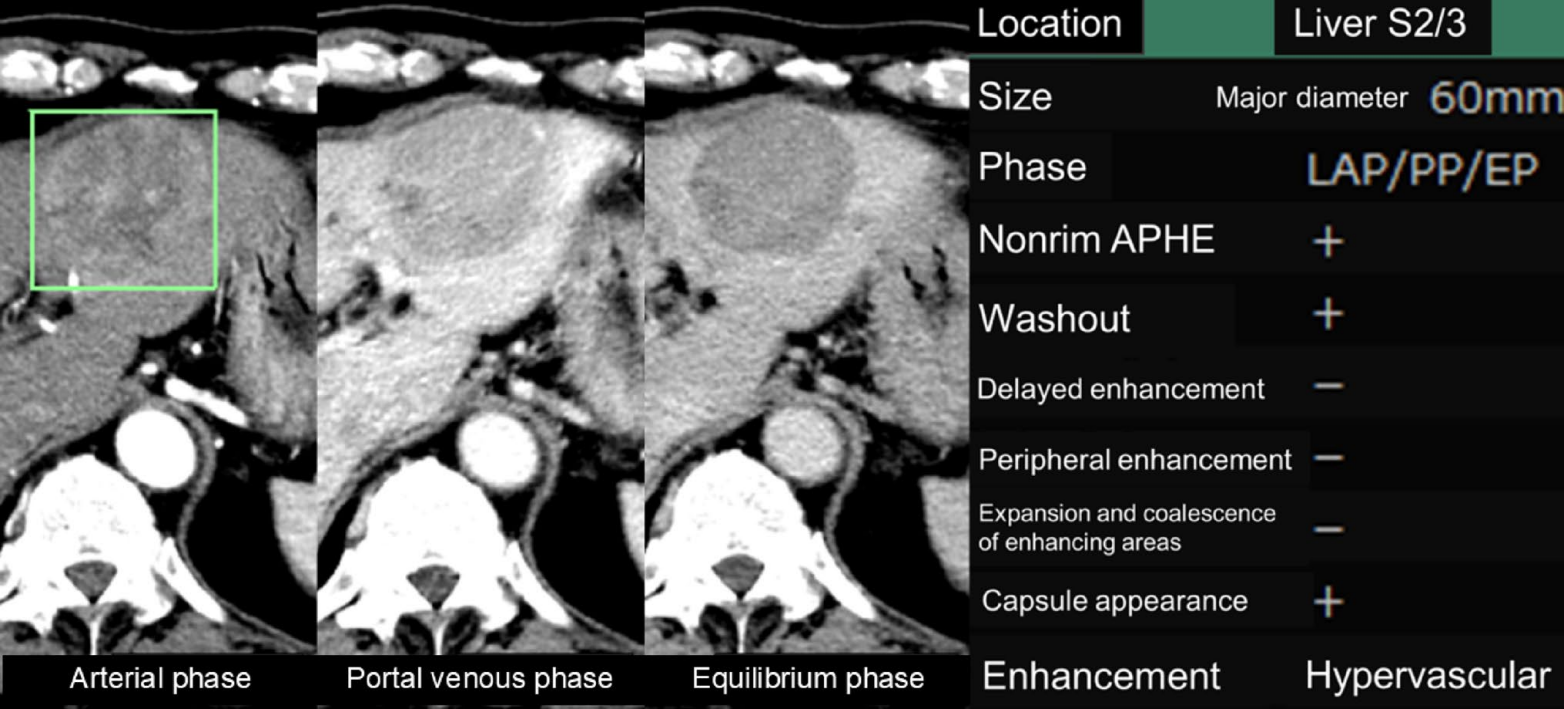
Example of commercially available software used for analyzing hepatic lesion characteristics in multiphasic liver CT imaging. A 60-mm tumor was identified in the lateral segment and diagnosed as HCC. AI analyzes voxel data from the arterial, portal, and equilibrium phases, providing imaging features such as tumor size, APHE, washout, capsule presence, and other characteristics. Radiologists can utilize these outputs to aid in making more objective and detailed imaging diagnoses (SYNAPSE SAI Viewer; FUJIFILM Corporation, Tokyo, Japan). This software has been approved for clinical use by Japanese regulatory authorities but not by the FDA. *APHE* Arterial phase hyperenhancement, *CT* Computed tomography, *HCC* Hepatocellular carcinoma

In addition to experimentally developed AI systems, commercially available software for assisting in the characterization of liver tumors has recently become available. Some of the authors evaluated the impact of a commercially available software tool (SAI Viewer; FUJIFILM Corporation, Tokyo, Japan) on the diagnostic performance of radiologists. This tool is not FDA-approved but has been authorized for clinical use by regulatory bodies in Japan. This study focused on how the software affects the ability of radiologists to evaluate hepatic lesion characteristics and differentiate tumor types using multiphasic liver CT (Nishigaki D, et al. Performance of radiologists in characterizing and diagnosing hepatic lesions using dynamic contrast-enhanced CT with and without artificial intelligence. Presented at RSNA 2024, Scientific Paper). The AI system processes voxel data from the arterial, portal venous, and equilibrium phases, providing imaging features such as tumor size, arterial phase hyperenhancement (APHE), washout, capsule presence, and other diagnostic indicators. Radiologists may use these outputs to support a more objective and detailed imaging diagnosis (Fig. 3).

Beyond AI applications targeting CT, attempts are also underway to apply AI to characterize liver tumors via MRI. Researchers developed a CNN system to classify seven types of focal liver lesions—cyst, hemangioma, focal nodular hyperplasia, other benign nodules, HCC, metastatic malignant tumors, and primary hepatic malignancies other than HCC—using multiphasic contrast-enhanced MRI and clinical data [22]. The system demonstrated performance comparable to that of experienced radiologists in liver tumor classification.

**Fig. 4:**
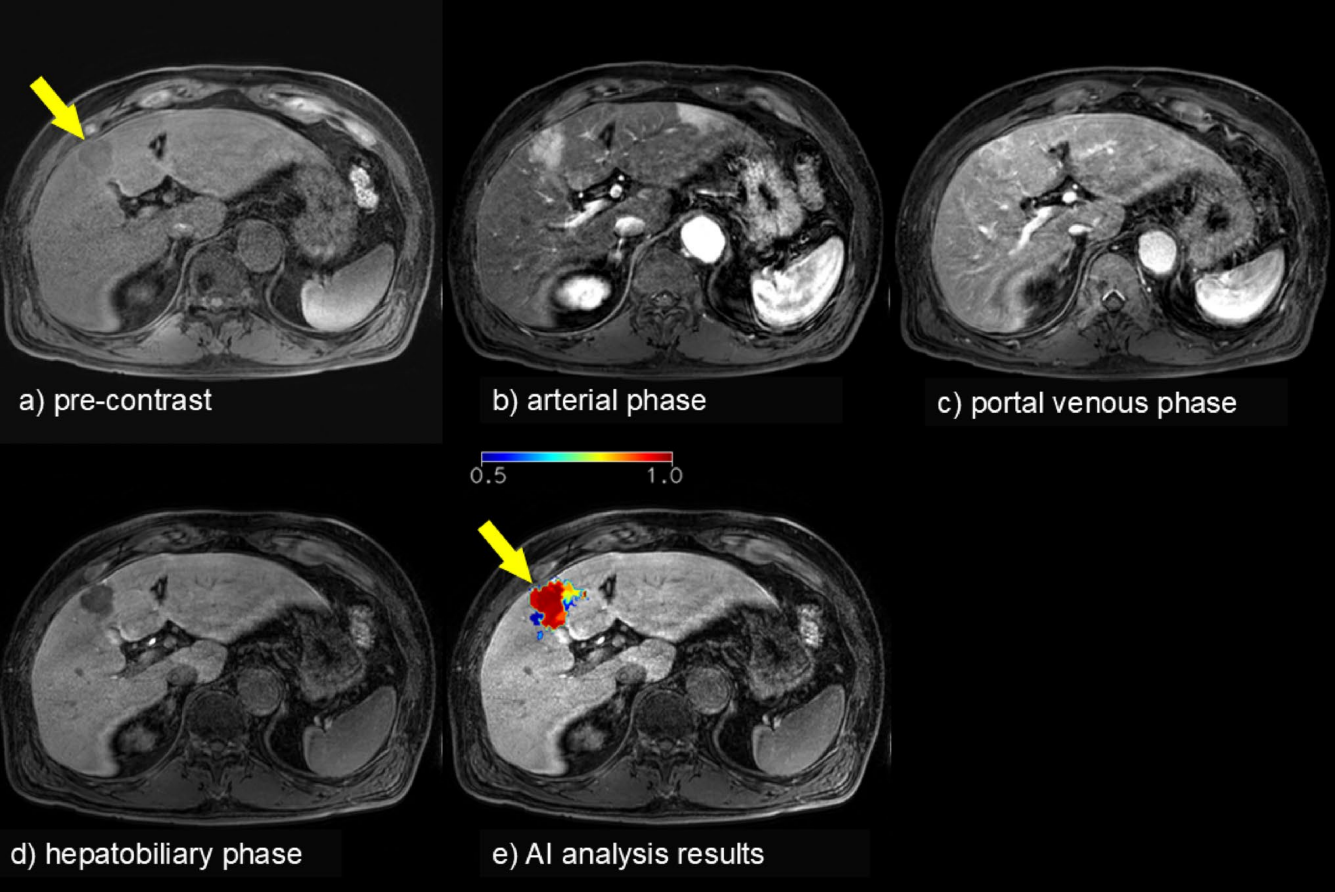
Example of a process using Gd-EOB-DTPA-enhanced MRI reading support software with AI. A 35-mm tumor is identified in the medial segment (**a**: arrow). This hypervascular tumor was diagnosed as HCC. The MRI reading support software (Cal.Liver.Lesion; Bayer Yakuhin Ltd., Osaka, Japan) quantitatively scores the degree of signal variation compared to surrounding tissue. The score, displayed in color, is overlaid on the hepatobiliary phase image, where the HCC region exhibits high values (red) (**e**: arrow). Although not FDA-approved, the software received regulatory approval for clinical use in Japan; however, it has been discontinued as of 2025. Assistance from this AI software could enhance the accuracy of MRI-based liver tumor diagnosis. *AI* Artificial intelligence, *Gd-EOB-DTPA* Gadoxetic acid, *HCC* Hepatocellular carcinoma, *MRI* Magnetic resonance imaging

Gadoxetic acid (Gd-EOB-DTPA)‒enhanced MRI is widely used in liver imaging, and it is well known for its high diagnostic accuracy in liver tumor detection [23]. However, interpreting these images requires specialized expertise, which can be a barrier in clinical practice. Therefore, there is a growing need for AI systems that can assist non-expert physicians in interpreting contrast-enhanced MRI scans. In 2023, Japan approved an AI-based MRI interpretation software (Cal.Liver.Lesion; Bayer Yakuhin Ltd., Osaka, Japan). Although not FDA-approved, the software received regulatory approval for clinical use in Japan; however, it has been discontinued as of 2025. While such AI tools are not yet widely adopted, the use of such tools may enable physicians unfamiliar with liver MRI to appropriately interpret the images, thereby contributing to improved patient management (Fig. 4).

There is strong interest in developing AI systems that can simultaneously detect and characterize liver tumors, as an alternative to those that perform these tasks separately. A Recent large-scale study has highlighted the value of such integrated models in radiological assessment of focal hepatic lesions [24]. The Liver Artificial Intelligence Diagnosis System (LiAIDS) is an end-to-end AI system designed for simultaneous detection and characterization of focal liver lesions based on contrast-enhanced CT and patient clinical data. Trained on a large-scale, multicenter dataset (12,610 patients, 18 hospitals), it achieved F1-scores of 0.940 for benign and 0.692 for malignant lesions. The system improved radiologists’ diagnostic performance across all levels of experience. In a triage cohort of 13,192 patients, it accurately classified 76.46% as low risk (negative predictive value: 99.0%). Its robustness and generalizability across imaging platforms support its integration into clinical workflows.

### Segmentation using AI

Segmentation involves identifying and dividing regions of interest (such as organs, anatomical segments, or lesions) within an image. In liver imaging, segmentation can be used to measure liver volume. Usage of 3D volume data improves measurement accuracy compared to that using conventional thick-slice images [25]. Therefore, automatic segmentation of the liver in 3D data enables simple, accurate liver volume measurement, which is essential for preoperative evaluations and holds significant clinical value.

**Fig. 5:**
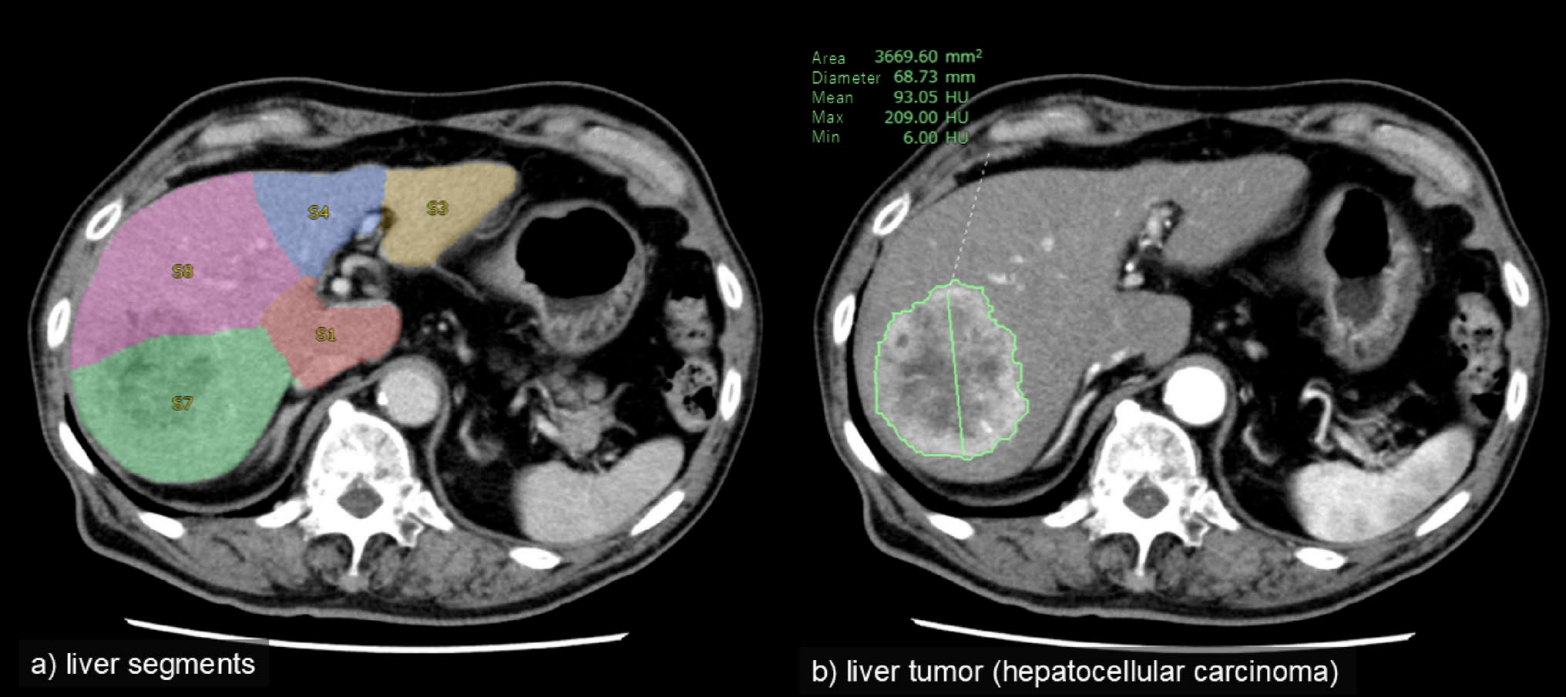
Segmentation using an AI-equipped radiological viewer. Radiological viewer with integrated AI features (SYNAPSE SAI viewer, Fujifilm; approved for clinical applications in Japan, not FDA-approved) provides reading support functions. **a** Automatic segmentation of liver segments, which is displayed as an overlay on CT images. **b** Manual input of two points on the margin of a liver tumor to extract the tumor region. This automated/semi-automated segmentation is useful for pre-treatment and post-treatment assessments. *AI* Artificial intelligence, *CT* Computed tomography

AI-based automatic segmentation produces liver volume measurements closely matching those of manual methods, with high reproducibility and reduced processing times [26]. Identifying hepatic segments containing tumors is critical for pre-treatment assessment. Recently, several radiological imaging viewers have integrated AI to automate liver segment segmentation (Fig. 5a).

AI has also been applied to automatically segment tumors [27]. Commercially available image viewers with these features also exist (Fig. 5b). For metastatic liver tumors, AI-based automatic segmentation improves accuracy compared to those of conventional methods such as Response Evaluation Criteria in Solid Tumors (RECIST) 1.1 [28].

However, several significant challenges can substantially affect the accuracy of AI-based liver segmentation, including imaging artifacts, anatomical distortions, and large hepatic tumors. Common sources of artifacts include respiratory motion, cardiac motion, and the presence of metallic objects. When such artifacts are present, they can degrade segmentation accuracy in both manual and automated approaches. To mitigate these artifacts, correction techniques have been developed [29–31], which can be expected to enhance the performance of automated segmentation. Additionally, inter-patient variability in liver shape and changes across respiratory phases can also negatively impact segmentation accuracy. Massive hepatic tumors further contribute to shape distortion, posing another obstacle to accurate segmentation. Addressing these issues remains an important direction for future research.

### Radiomics

**Fig. 6:**
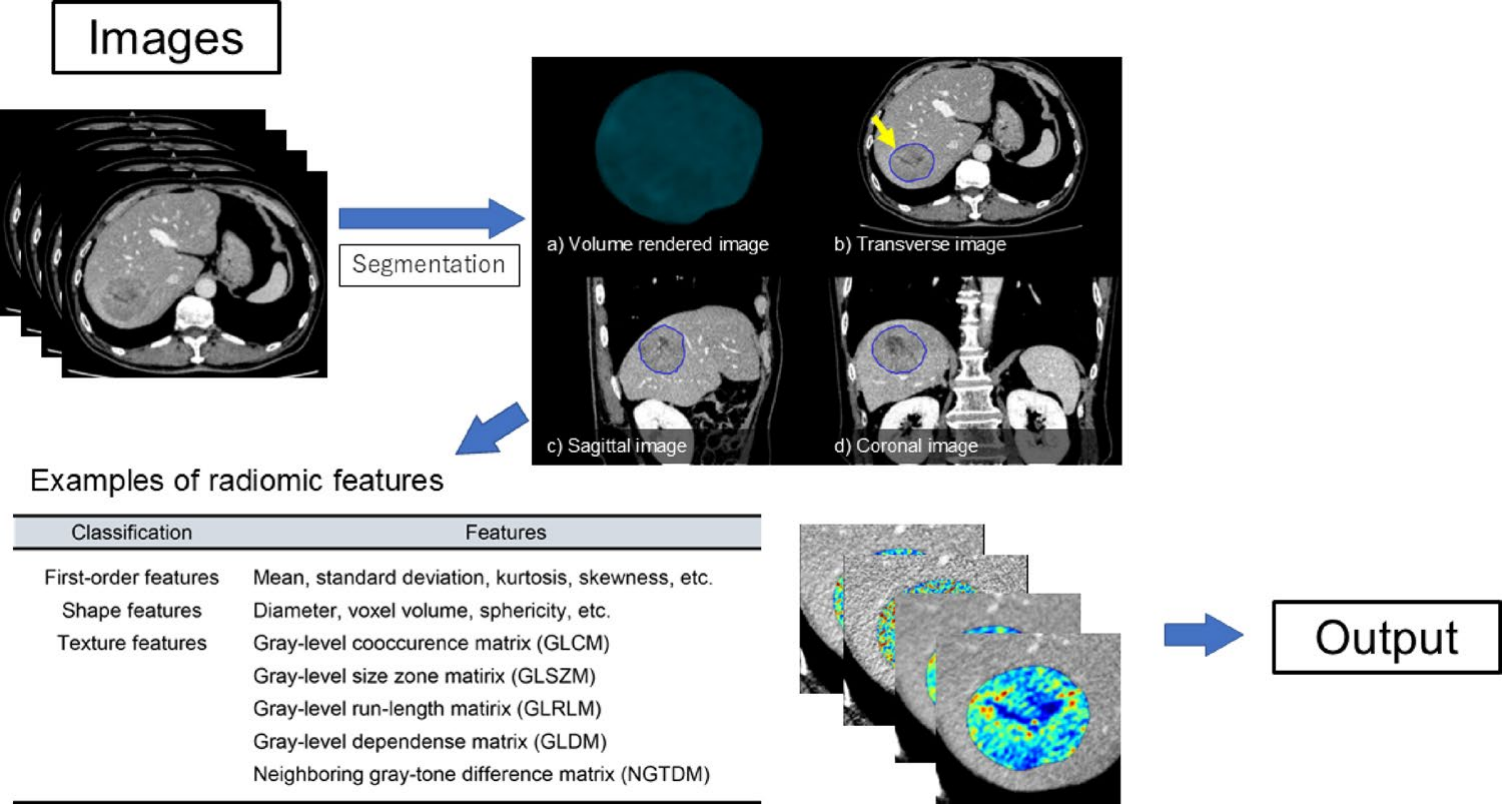
Schematic representation of radiomics workflow. Segmentation of the VOI is first required for analysis. Manual, semi-automated, or fully automated segmentation can be used. Subsequently, several radiomic imaging features are calculated. Finally, output, such as the probability of 5-year survival, is generated. * VOI* Volume of interests

Radiomics involves extracting numerous quantitative features from medical images (Fig. 6) [32]. In liver imaging, radiomics has been applied to tumor diagnosis [33], prediction of microvascular invasion in HCC [34], prediction of CD73 expression in colorectal cancer liver metastases [35], prognostication in HCC [36], and prediction of treatment outcomes in HCC following transarterial chemoembolization (TACE) [37, 38], surgical resection [39], and radiation therapy [40, 41], as well as following neoadjuvant therapy for colorectal liver metastases [42].

As an example of its use in tumor diagnosis, a previous study demonstrated that tumor screening based on radiomics features from contrast-enhanced CT has been shown to be feasible, enabling noninvasive classification of liver tumors and healthy tissue and supporting their role as potential imaging biomarkers [33]. Radiomics features from preoperative CT have also been used to predict MVI in HCC. A hybrid model combining radiomics and clinical data achieved high performance (AUC up to 0.86) and enabled risk stratification for recurrence and survival, supporting its role in preoperative decision-making [34]. Radiomics-based prediction of CD73 expression in colorectal cancer liver metastases has also been investigated. An Attentive Interpretable Tabular Learning (TabNet) model trained on preoperative CT images yielded high accuracy (AUC up to 0.79) and a radiomic score that correlated with histologic CD73 expression and independently predicted recurrence and survival [35].

In the context of prognostic tumor staging, radiomics features extracted from baseline MRI have been shown to improve prognostication in HCC. When combined with the Barcelona Clinic Liver Cancer (BCLC) staging system, radiomics enhanced the prediction of transplant-free survival, outperforming either method alone. These results highlight the potential of radiomics as a complementary tool for risk stratification [36]. An increasing number of studies have demonstrated the value of radiomics for post-treatment outcome prediction. A radiomics model incorporating whole-liver MRI features and clinical data has shown high accuracy in predicting survival in HCC patients undergoing continued TACE after refractoriness [38]. As an example related to surgical resection, a contrast-enhanced CT-based radiomics nomogram incorporating radiomics features, neutrophil-to-lymphocyte ratio, and alpha-fetoprotein (AFP) showed strong performance in predicting overall survival after radical hepatectomy in HCC patients [39]. The model showed good prognostic performance and outperformed traditional staging systems. Regarding radiation therapy, a machine learning model combining pre-treatment MRI-based radiomics features with clinical parameters and AFP showed improved performance in predicting HCC response to Yttrium-90 radiation segmentectomy, compared to clinical data or AFP alone [41]. For colorectal liver metastases treated with neoadjuvant therapy, a machine learning model using CT-based radiomics features from tumoral and peritumoral regions outperformed radiologist assessments based on RECIST 1.1 and morphologic criteria in estimating pathologic treatment response [42].

Commonly extracted radiomic features include shape features (e.g., diameter, volume), first-order features (e.g., mean, standard deviation), and texture features derived from the spatial distribution of pixel values [32]. The total number of extracted features can exceed hundreds. To select the most relevant features and avoid overfitting, dimensionality reduction techniques are applied. A commonly used method is the least absolute shrinkage and selection operator (LASSO), which performs both variable selection and regularization to enhance prediction accuracy [43]. Random forests, another widely used algorithm, are ensemble learning methods that construct multiple decision trees and output the class that is the mode of the classes from individual trees [44]. These algorithms are frequently employed in radiomic analyses for classification, regression, and feature importance ranking. Additionally, instead of relying on predefined features as mentioned above, deep learning has been explored to discover features automatically. Therefore, machine learning plays a crucial role in radiomics.

Despite the growing body of research, however, its clinical adoption remains limited [45]. One major challenge is the issue of repeatability and reproducibility [46]. Radiomics workflows involve numerous variables, including imaging equipment, acquisition parameters, reconstruction algorithms, segmentation methods, and feature extraction software. The vast number of possible combinations makes it difficult to ensure that results obtained under one condition can be reproduced under another. A phantom and human study found that only a subset of radiomic features were both repeatable and reproducible across scanners and acquisition settings [47]. In that study, wavelet and Laplacian of Gaussian features demonstrated the highest stability, highlighting the importance of robust feature selection in radiomics modeling. Another challenge is the lack of standardization. The guidelines include several recommendations aimed at standardizing radiomics practices; notably, they advise the use of software tools that adhere to the Image Biomarker Standardisation Initiative (IBSI) guidelines, such as PyRadiomics, for feature extraction [45]. A further limitation is that most radiomics studies are retrospective in nature, often resulting in low levels of evidence. For radiomics to be successfully integrated into clinical practice, it is essential to generate findings supported by standardized methodologies, account for repeatability and reproducibility, and provide high levels of evidence.

### Challenges and prospects of AI in evaluating liver tumors

Despite significant progress, several challenges persist in developing and implementing AI. A major challenge in building AI models is the lack of large, annotated datasets provided by radiologists. This is particularly important because the quality of training data substantially influences both the performance and generalizability of AI systems. One strategy to alleviate the annotation burden is to reuse information from medical records collected during routine clinical practice. Several researchers demonstrate that extracting disease information from radiological diagnostic reports and using it as training labels eliminates the need for additional manual annotation [48].

Another challenge in clinical AI applications is the “black-box” nature of these systems, which can lead to challenges such as the risk of unintended or inappropriate outputs and the difficulty in assessing the reliability of AI results when the underlying rationale is not clear. A promising solution to these challenges is the implementation of explainable AI (XAI), which enables AI to provide explanations for their outputs, allowing human users to understand the reasoning behind them [49–51]. While research in this field remains limited, it is expected to grow in importance. In XAI, techniques such as saliency maps, gradient-weighted class activation mapping (Grad-CAM), local interpretable model-agnostic explanations (LIME), and Shapley additive explanations (SHAP) are widely used. These methods aim to elucidate the rationale behind model predictions and have become increasingly prevalent in medical image analysis. Although these visual explanation techniques can highlight image regions relevant to model outputs, they do not necessarily reflect the reasoning processes that radiologists employ when interpreting images. Another important approach involves generating explanations for AI outputs using natural language, without relying on saliency maps or similar visual methods. Several researchers have contributed to the development and evaluation of an XAI system designed to aid in differentiating liver lesions in Gd-EOB-DTPA-enhanced MRI using Liver Imaging Reporting and Data System (LI-RADS) language (Zhang C, Jin Z, Hori M, et al. AI-aided diagnostic system providing explanations in LI-RADS language in liver cancer diagnosis using MRI. Presented at RSNA 2024, Scientific Paper). The implementation of XAI may significantly facilitate the clinical adoption of AI in liver tumor evaluation by enhancing model transparency, which is critical for building trust among radiologists and supporting decision-making in high-stakes clinical settings.

Additional challenges should also be considered. Most studies are retrospective in nature, which often leads to low levels of evidence. Furthermore, many rely on relatively small datasets, potentially limiting the generalizability of AI performance. Additionally, most existing approaches focus on a single modality. The development of multi-modal AI systems that integrate CT, MRI, electronic medical records, biomarkers, and treatment history is essential.

## Conclusions

The role of AI in liver tumor imaging has been discussed and its significance continues to grow. AI-based image reconstruction is widely used in clinical practice, providing benefits such as improved image quality and reduced radiation exposure. AI-assisted diagnostic software is less common in liver imaging compared to other areas, such as the lungs; however, its application is gradually increasing and is expected to become an integral part of routine clinical practice. Advancement in emerging technologies (such as radiomics, currently at the research stage) is expected to have broader applications in image assessment, treatment planning, treatment evaluation, and prognosis prediction. Moreover, recent developments in generative AI, including large language models such as ChatGPT, show significant promise for potential applications in medical imaging.

To promote broader clinical integration, future studies should address key issues such as the repeatability and reproducibility of radiomics, which depend on consistent imaging acquisition settings and standardized feature extraction methods; the development of multimodal AI systems that incorporate imaging, clinical, and biomarker data; the construction of large, diverse annotated datasets for robust training and validation; and the implementation of XAI techniques to improve interpretability, user confidence, and responsible deployment in routine clinical settings.

## Data Availability

No datasets were generated or analysed during the current study.
